# Femtosecond Laser Axotomy in *Caenorhabditis elegans* and Collateral Damage Assessment Using a Combination of Linear and Nonlinear Imaging Techniques

**DOI:** 10.1371/journal.pone.0058600

**Published:** 2013-03-06

**Authors:** Susana I. C. O. Santos, Manoj Mathew, Omar E. Olarte, Sotiris Psilodimitrakopoulos, Pablo Loza-Alvarez

**Affiliations:** 1 ICFO-Institut de Ciencies Fotoniques, Mediterranean Technology Park, Castelldefels (Barcelona), Spain; Brown University/Harvard, United States of America

## Abstract

In this work highly localized femtosecond laser ablation is used to dissect single axons within a living *Caenorhabditis elegans* (*C. elegans*). We present a multimodal imaging methodology for the assessment of the collateral damage induced by the laser. This relies on the observation of the tissues surrounding the targeted region using a combination of different high resolution microscopy modalities. We present the use of Second Harmonic Generation (SHG) and Polarization Sensitive SHG (PSHG) to determine damage in the neighbor muscle cells. All the above is done using a single instrument: multimodal microscopy setup that allows simultaneous imaging in the linear and non-linear regimes and femtosecond-laser ablation.

## Introduction

Nowadays lasers are one of the most powerful and versatile tools in the biomedical field. Apart from their use in macro and microscopic imaging techniques, their application in the non-invasive modification of biological samples is of particular importance. For these purposes, ultrashort pulsed lasers employing near infrared wavelengths have been shown to be the ideal tool when a very controlled and precise modification is required. These lasers have the ability to induce nonlinear photoionization and thereby to confine interaction to the focal volume (<1 femtoliter) of a tightly focused beam [1]. This enables controlled incisions with submicrometer precision can be induced. In addition to this, the use of near infra-red (NIR) light provides much higher tissue penetration due to a reduced (linear) absorption and scattering of the sample. This is opening up a whole new window for laser nanosurgery as a non-invasive powerful surgery technique that can be applied in a vast range of biomedical areas.

Although the precision of the femtosecond laser scalpel in biological samples is widely accepted to be superior to other surgical techniques, the complex nature of the interaction light-sample calls for stringent tests on how precise this tool is. A logical way to perform these tests is to “observe” how much damage is sustained in the structures surrounding the target of surgery and, specially, in a specific biological sample. Assessment of this collateral damage is not just important to validate the precision of the surgical tool but it would also help to understand how a specific biosample responds to the surgery (chemically, structurally and behaviorally). Such determination of collateral damage is not trivial when the surgery is performed at the microscopic or submicroscopic level. Ordinary transmission light microscopes are not optimal for a detailed characterization of the resultant low-contrast subcellular modifications. For this reason, researchers have opted for the use of contrast enhancing microscopy techniques to observe and characterize the effects of laser irradiation. Among them, fluorescence microscopy has been particularly useful to monitor the laser induced damage to the cells, either by using a fluorescent reporter of the chemical species leading to cell degradation (*e.g*. reactive oxygen species ROS[2]) or by following the rise in auto fluorescence signal in the surroundings of the dissected area [3]–[4].

A very interesting case of the use of femtosecond lasers for subcellular surgery is the dissection of axons, or axotomy, of the nematode *Caenorhabditis elegans* (*C. elegans*). Single *C. elegans* neuron axons were first used as targets for laser surgery in a report by Yanik *et al.* [5]. In this study spontaneous axon regeneration was observed for the first time in this model organism. Moreover, individual axons of *C. elegans* were precisely cut with a focused femtosecond laser beam inducing an anomalous behavioral response that was restored to normal once the regeneration was completed. This technique allowed not only the study of the response of the organism to a single axon dissection but also a much more exhaustive study at different levels of neuronal interconnections and their particular regeneration properties [6]–[9].

**Figure 1:**
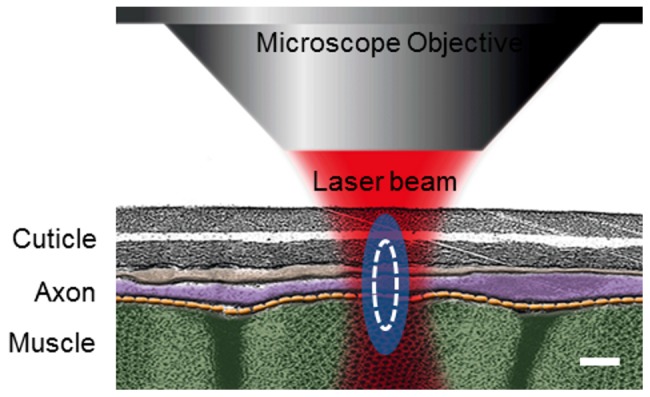
TEM illustration of the anatomical region of *C. elegans* where the laser axotomy is performed . Body wall muscles are shown in green, axons in purple and cuticle in gray. Blue ellipsoid is the estimated full-width-at-half-maximum of the beam's point spread function. White line represents the limit of the maximum plasma density generated at the focal spot. Scale bar 300 nm.

*C. elegans* D-type motoneurons are particularly attractive for the study of collateral damage induced during a femtosecond laser axotomy due to several reasons. First, these neurons extend axons that are extremely thin (few hundred nanometers) and reside in a complex environment surrounded by cuticle (above) and muscle (below) as can be seen in Figure 1. (background figure adapted from Wormatlas [10]). This means that even a very tiny mis-targeting or inaccuracy in the use of the laser surgery tool would show up as a collateral damage in the surrounding structures. Secondly, they provide an effective method towards the understanding of the triggered responses after axotomy that lead to axon regeneration [5]–[9]. Therefore, having a system that produces very precise cuts and has the capability to readily detect any unwanted/collateral damage is very important in the understanding of the mechanisms of neuronal regeneration.

So far, evaluation of collateral damage resulting from axotomy has been done by employing wide field fluorescence microscopy to measure the separation of the axon ending tips as well as the size of the damage in the muscle cell in close proximity to the axons. Both axons and muscles were labeled by green fluorescent protein (GFP) [11]–[12]. While this can provide an approximate indication of the size of the damaged areas, there is still the need for methods that would provide more detailed information about the extent and the nature of the collateral damage, as well as the many processes that could take place during the surgery. In a previous work, we showed how the combination of confocal fluorescence microscopy simultaneously with Laser-scanned Transmitted light microscopy (LT) provides a powerful tool not only for the real-time observation of the surgery with high resolution but also for the study of the different dynamical processes happening during the procedure [4]. Nevertheless, these linear microscopy techniques do not fully take advantage of the endogenous sources of contrast present in the region surrounding the operated axon. Particularly, the muscular structures of the *C. elegans* nematode have been shown to be sources of Second Harmonic Generation (SHG), a nonlinear optical effect that can be excited with ultrashort pulsed lasers [13]. In contrast to confocal and two-photon excited fluorescence (TPEF), which rely on photon absorption, SHG is a nonlinear scattering process and hence, in principle, it deposits no energy to the matter with which light interacts. The energy conservation and label-free characteristics enable minimally invasive imaging, which is highly desirable especially for *in vivo* studies. The most widely studied biological structures in tissues that produce endogenous SHG signal are based on collagen, muscle, and microtubules elementary structures. Furthermore, these elementary SHG scatterers, when excited with different incoming linear polarizations, provide a SHG response that is characteristic of the local scatterer orientation. This polarization dependent SHG (PSHG) technique has been used to interpret images taken from *C. elegans* muscles [14]. Furthermore, using PSHG imaging it is possible to determine the orientation, *θ_SHG_*, and degree of organization, *Δθ*, of the SHG elementary source (i.e. the hyperpolarizable molecule) inside the muscular tissue.

In this study we cut a set of axons of *C. elegans* D-Type motoneurons using femtosecond unamplified MHz pulses and provide a new approach for a rigorous, detailed and more precise assessment of the collateral damage induced to the surrounding tissues. We present the use of a combination of linear and nonlinear high-resolution imaging techniques, all of them integrated in a single multimodal optical workstation, to monitor any change induced to the tissues around the operated axon [15]. Damage to the cuticle produced with the femtosecond laser can be readily observed with LT. At the same time, confocal microscopy is used to reveal minute collateral tissue damage that generates autofluorescence or produces any change in the original fluorescence of the structures. Finally, previously published laser axotomy studies were relying on genetically modified worms expressing muscle fluorescence to assess the caused damage in this tissue [11]. In this paper we exploit the advantages offered by SHG to extract information of unlabeled muscular tissue. Furthermore, we present PSHG as a novel high-resolution contrast mechanism able to reveal laser axotomy inflicted molecular damage in the muscle of this model organism.

## Materials and Methods

### The multimodal microscope

A multiphoton microscope was built around a commercial confocal (Nikon C1–Si) inverted microscope (Nikon Eclipse Ti–E [14], Nikon Inc., Japan) using a Kerr-lens mode-locked Ti:Sapphire oscillator (Coherent MIRA 900f) producing 150 fs (FWHM) pulses at a repetition rate of 76 MHz. This microscope incorporate two independent pairs of galvanometric mirrors, one for continuous-wave visible laser (within scan head of Nikon C1–Si) and the other (placed externally) for the ultrashort pulsed laser. Both laser sources were coupled through 2 different input ports in such a way that both the confocal and multiphoton sections could work independently and simultaneously. This multimodal unit could, therefore, be used in the linear and nonlinear regime with a variety of different techniques working in a simultaneous way. In particular, the nonlinear input could be used for performing the axotomy while the confocal unit could be used to visualize axotomy and the collateral damage at the same time. For more details on the full capabilities of this set-up please see Mathew *et al*. [15].

### Worm mounting

*C. elegans* were grown on nematode growth medium (NGM) agar plates using standard procedures [16]. To visualize the axons and to perform most of our axotomy studies we employed the transgenic strain juIs76 [unc-25::GFP] II that expresses GFP in D-type motoneurons. To confirm that SHG gives an equivalent/superior muscle damage information, when compared to fluorescence based methods, we employed the transgenic strain RP1: trIs10 [myo−3p::MB::YFP+myo−2p::YFP+ceh−23::HcRed+unc−25::DsRed+unc−129nsp::CFP] that expresses YFP (membrane anchored) in muscle cells. In both cases, the worm cultures were synchronized and single L4 *larvae* were mounted on 2% agar pads with 0,8 µl of 10 mM Levamisole (Dr. Ehrenstorfer) between two 170 µm glass slides. These were sealed with melted paraffin for sample stabilization.

### Axotomy

Axotomies were performed on the commissures of the D-type motoneurons on the most ventral/dorsal part of the axon just after its association with the cords (∼1–5 µm away). The position of the precise focal plane for cutting was found based on simultaneous TPEF and SHG imaging of the axon and the body wall muscles, respectively. This provided us with the anatomical references to finely adjust the position of the laser beam precisely at the axial center of the axon (see Figure 1). A different number of axotomies per worm (in different axons) were performed resulting in a total of sixty-one surgeries. Axotomies were performed by parking the tightly focused laser beam on the target point while controlling the irradiation time with an electronic shutter. For this work, the laser central wavelength was set to 868 nm in order to optimize the imaging performance the system, without compromising the available laser power required for ablation. We employed an oil immersion 60×1.4 NA microscope objective that focuses the beam to a point spread function (PSF) of full-width-at-half-maximum (FWHM) dimensions of 0.45 µm transversal and 1.2 µm axial [17]. To find the appropriate laser power for cutting, the exposure time was set to 200 ms and the laser power was increased up to the value that led to an effective disruption of axons. This methodology yielded an optimal average power of 90 mW (energy per pulse of 1.2 nJ) which corresponds to a peak intensity of approximately 3×10^12^ W/cm^2^at the sample plane. The same power was used throughout the experiments.

### Multimodal imaging for collateral damage assessment

It is important to determine if the axons are indeed severed and not simply photobleached and appear as a cut at the point of laser interaction (point of surgery). The following cues helped in determination of successful axotomy: a) retraction of the axon; b) sealing and/or formation of bumps at the point of laser surgery and c) spilling of axoplasm [4] [12]. Moreover, in order to confirm complete severing, we performed several axotomies on GFP labeled ASJ neurons followed by DiI staining (data not shown) [18].

The presented collateral damage assessment methodology relies on the visualization of the tissues surrounding the dissected axon. The following collateral damage scenarios can be detected: a) Damage to the cuticle is easily observable form the laser-scanned transmitted light microscopy (LT) images; b) Any collateral tissue damage, both in the cuticle or in the muscle can potentially be detected by looking out for localized increase in autofluorescence around the point of laser interaction using the confocal microscopy images; c) to have an even better determination of the collateral damage to muscle, SHG microscopy of the muscle tissues could be employed. It may be noted that all the three imaging modalities (LT, confocal and SHG) is done using the same instrument that performs the laser surgery.

In the following description we present the methodology used for the damage assessment. First, the area surrounding the point of surgery was imaged pre-surgically using TPEF and SHG microscopy. The axotomy was then performed using custom-made software that allows us to select the point on the axon to be dissected using the TPEF image. The surgical procedure was recorded while being performed using confocal and LT microscopy to help observe the effective dissection of the axon as well as any dynamical effect that provide information about collateral damage. Immediately after the surgical procedure, a second set of nonlinear (TPEF and SHG) images were taken, at several focal planes around the point of surgery, to correct for any changes in the focal plane over the tissue relaxation time. Finally, worms presenting axotomies that do not show clear collateral damage were selected to be imaged with PSHG microscopy [19]. In this case if there is any change in the myosin structure caused by the laser tissue interaction the PSHG technique could potentially reveal it.

In order to characterize the level of damage that can be reported with the PSHG technique, a series of experiments using worms expressing YFP in the body wall muscles were used (9 worms). This enabled to observation of fluorescence, SHG and PSHG images from the same axotomized Region of Interest (ROI).

## Results

**Figure 2:**
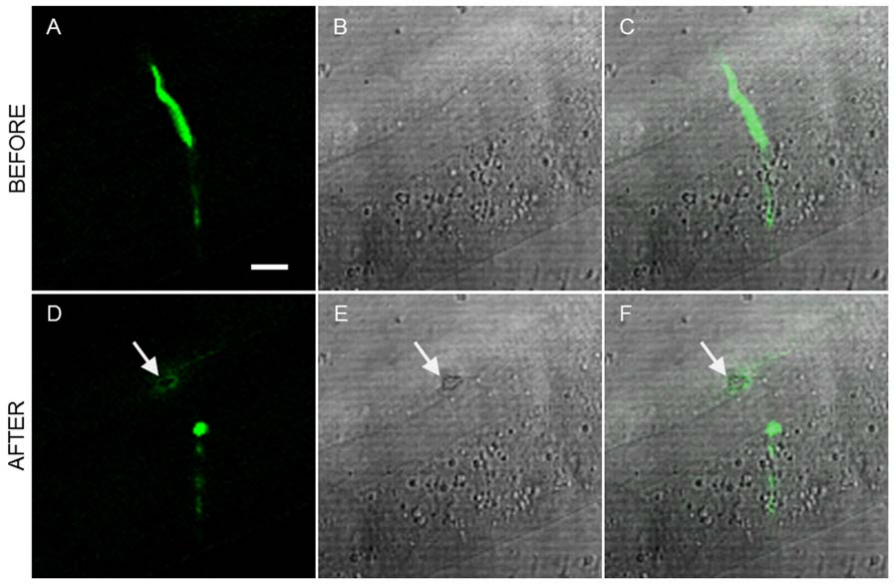
Damage assessment using linear imaging techniques. a) Confocal, b) LT and c) combined images of the region surrounding the axon before the laser dissection. d–f) show the same region after the surgery (see Media S1). Damage is evidenced by increased autofluorescence in the confocal image and a dark spot in the LT image. Both damage structures colocalize at the combined image. Excitation of the GFP labeled neurons was done at 488 nm. Arrows point to the place of the laser axotomy. Scale bar 10 µm.

Sixty-one axons were laser cut and simultaneously imaged using confocal microscopy. Fifty six of the 61 operated commissures were successfully severed. The inflicted collateral damage was determined using different imaging techniques using our multimodal optical workstation. Figure 2 shows the typical example of a laser axotomy and the high resolution imaging approach for the visualization of collateral damage. Figures 2 (a–c) and (d–f) show confocal and LT images before and after the axotomy, respectively. The post-surgical confocal image, depicted in Figure 2(d), reveals a circular pattern of autofluorescence that is generated around the point of surgery. This indicates that some collateral damage is induced by the laser to the close-by tissue. In addition, this damage is also evidenced by a clear retraction of the axon tips in the laser axotomy region [4]. Moreover, the dark spot evidenced in the post-surgical LT image (in Figure 2(e)) clearly indicates that the damage was induced in the cuticle. As expected, both observed damage patterns colocalize over the same region as can be seen in Figure 2(f).

**Figure 3:**
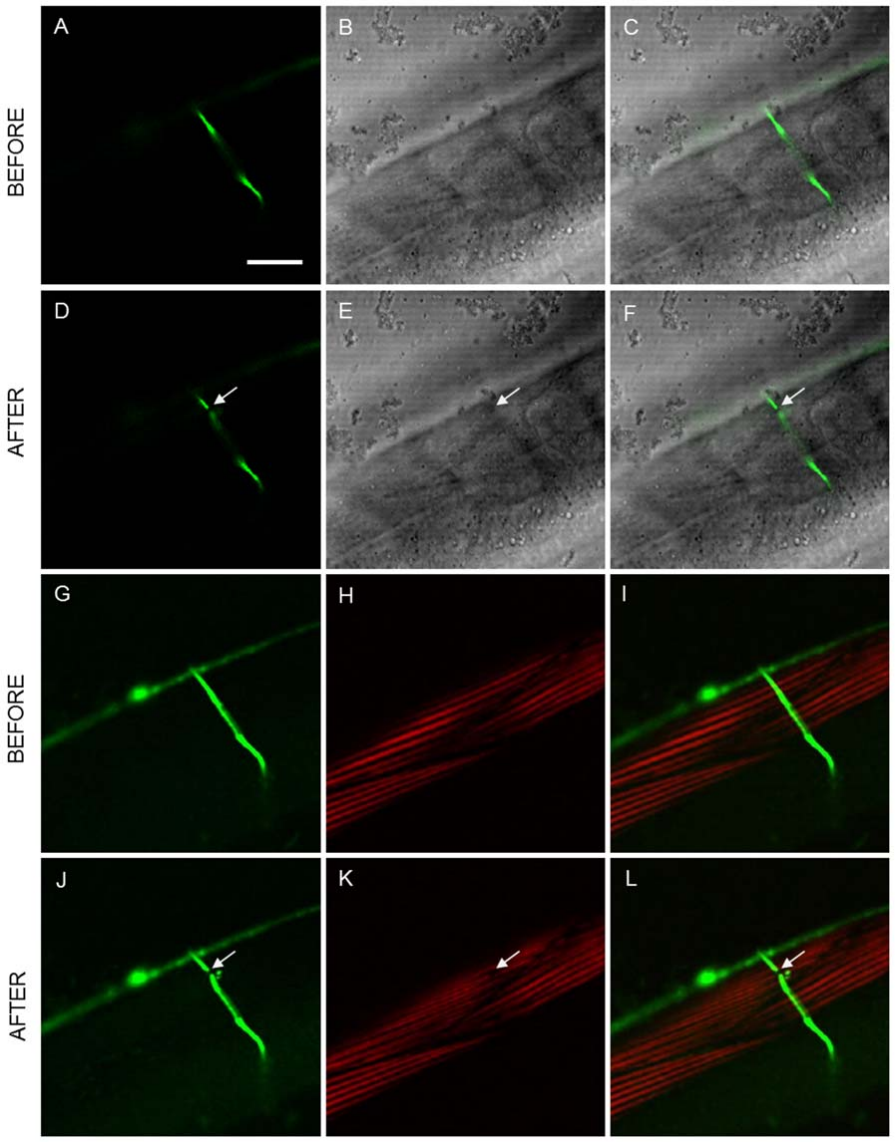
Collateral damage assessment using linear and nonlinear imaging techniques. Linear: a) confocal, b) LT and c) combined images of the region surrounding the axon before the laser dissection; d–f) show the same region after the surgery (Media S2). Nonlinear: g) TPEF, h) SHG and i) combined images before the laser dissection; j–l) show the same region after the surgery. No collateral damage was observed in CFM while damage in muscle is evidenced with SHG microscopy. Arrows point to the place of the laser axotomy. Scale bar 20 µm.

Post-surgical nonlinear imaging using combined simultaneous TPEF and SHG microscopy further aided in determination of collateral damage. Figure 3 shows complete multimodal imaging performed at the region closer to the axotomy. Figures 3 (a–c) and (g–i) show the multimodal images taken before axotomy, whereas Figures. 3 (d–f) and (j–l) are the resulting images after the procedure. In this case, no collateral damage is observed either in the confocal image (Figure 3(d)) or in the TPEF image (Figure 3(j)). Minimum collateral cuticle damage is observed in the LT image (Figure 3(e)). The surgical tool has made an efficient cut in the axon with minimum damage to the cuticle, however, changes have been induced to the muscle as can be observed in the Figure 3(k): reduction of the SHG signal in a small region around the dissection point and transition from Single-Band to Double-Band (SB to DB) signal structure of the muscle sarcomeres. This clearly illustrates the power of multimodal imaging (Confocal+LT+TPEF+SHG) in making a thorough assessment of collateral damage.

**Figure 4:**
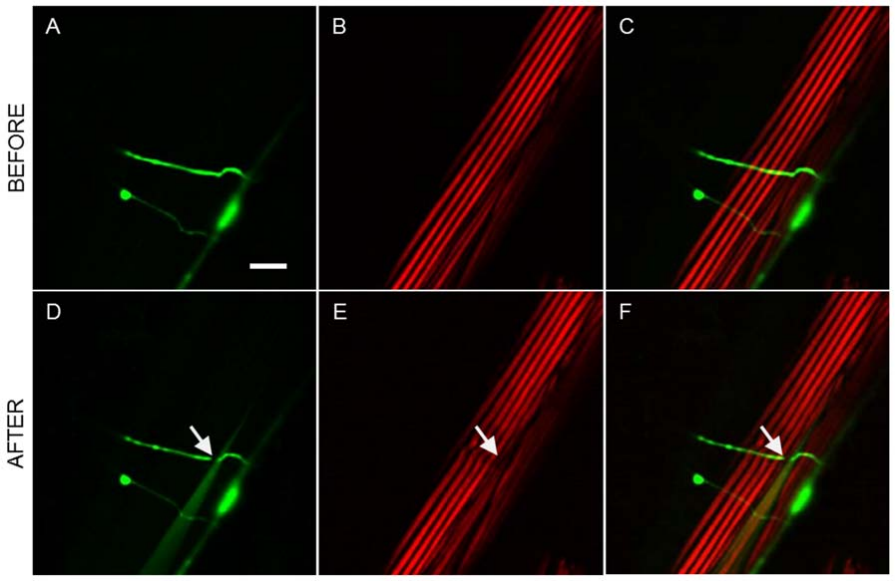
Laser-induced changes induced to the muscle visible with SHG imaging. a) TPEF, b) SHG, and c) combined images before the dissection; d–f) show the same region after the surgery. Arrows point to the place of the laser axotomy. Scale bar 10 µm.

There were a number of cases where no apparent collateral damage is observed using the linear and nonlinear fluorescent techniques. However, a slight “distortion” of muscle (not actual damage) could be observed in the SHG images of muscle. These distortions were in the form of either a minor reduction in the SHG signal from the muscles surrounding the region of surgery or a wavy-like appearance in the muscles. Such a case is shown in Figure 4 where, after axotomy, the muscle below exhibits wavy appearance without any actual loss of SHG signal in the muscle.

**Figure 5:**
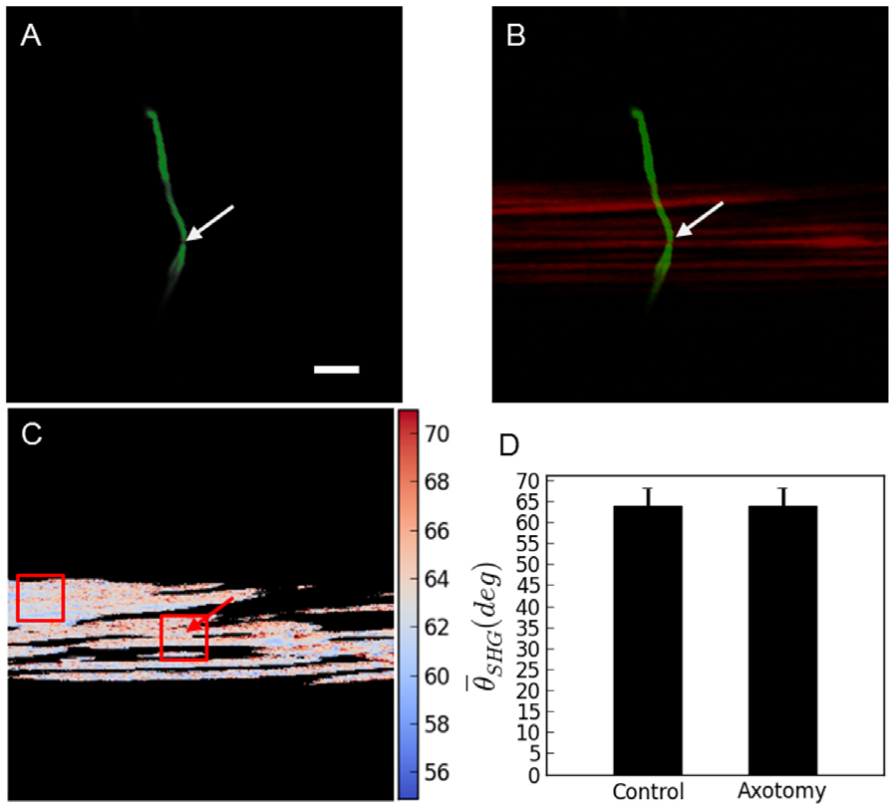
PSHG microscopy for the damage assessment after the axotomy. a) TPEF reveals a successfully cut axon. b) SHG signal from body wall muscle do not change after the surgery; c) Post-surgical pixel-resolution mapping of myosin *θ_SHG_* at the muscle obtained from PSHG (color bar in degrees); d) *θ_SHG_* (mean±1 standard deviation) for the muscles in the region surrounding the cut and in the control region (white squares). The control region was selected in the intact adjacent muscular cell far away from the axotomy. Unpaired two tailed *t*- test (n = 200 pixels for both sets) yields p>0.05 meaning that *θ_SHG_* mean is not significantly different between the two regions. Arrows point to the place of the laser axotomy. Scale bar 10 µm.

When the femtosecond laser tool is properly tuned and perfectly focused at the center of the axon incisions can be induced without any collateral damage. One such case (out of 12) is shown in Figures 5(a–b). TPEF evidenced that the axon is effectively severed whereas SHG image and PSHG analysis suggests that no damage was induced to the muscle. In these particular cases, where no damage was observed (with any of the above mentioned linear- and nonlinear imaging techniques), PSHG was performed to display the orientation of the hyperpolarizable molecule in myosin *θ_SHG_*. This was performed at a pixel level, as it can be seen in Figure 5(c). Unpaired two tailed *t*- test on these data shows that there are no significant differences in the mean value of *θ_SHG_* between the area surrounding the axotomy and the intact muscle well apart from the axotomy, Figure 5(d). This means that no damage could be detected, even at the level of the myosin structure of the muscles.

**Figure 6:**
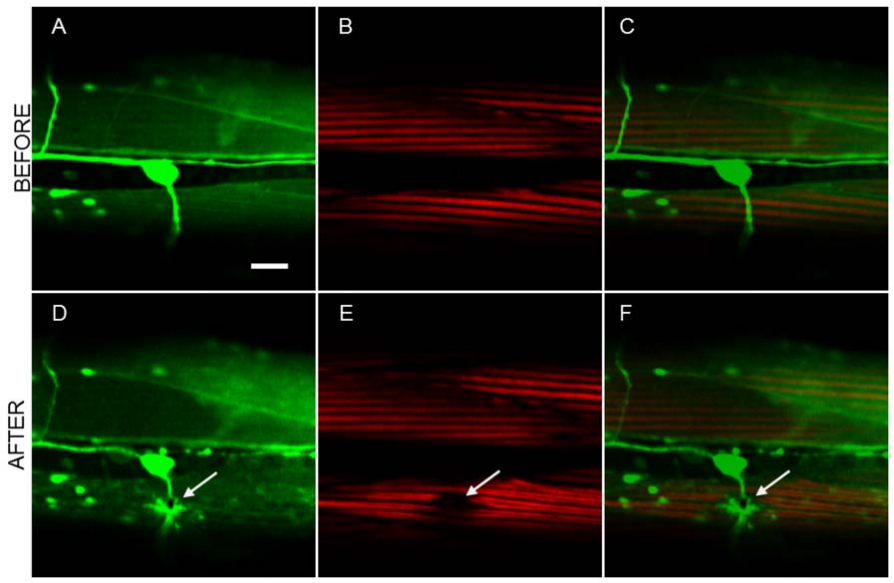
Comparison between SHG and fluorescence for the muscle damage assessment after the axotomy. Large collateral damage. Before the axotomy: a) TPEF images of YFP marked muscles and axons; b) SHG signal of the same muscles; and c) merge of TPEF and SHG images for comparison. Post-surgical images: d) TPEF and e) SHG images showing the laser damage hat is evident over a larger region. c) Shows a merged TPEF and SHG images for comparison. Arrows point to the place of the laser axotomy. Scale bar 10 µm.

**Figure 7:**
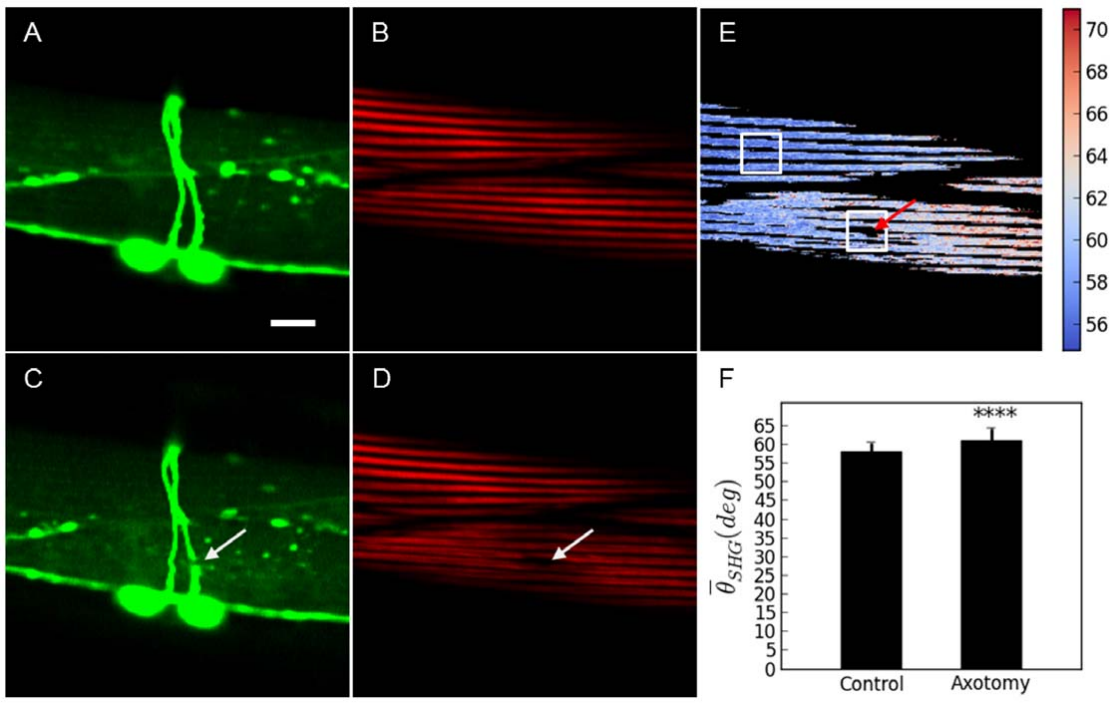
Comparison between PSHG and fluorescence for the muscle damage assessment after the axotomy. Medium collateral damage. Before the axotomy: a) TPEF image of YFP marked muscles and axons; b) SHG image of the muscles in the same region. After the axotomy: c) TPEF reveals a successfully cut axon showing a gap that interrupts the continuity of the axon. No other damage is apparent; d) SHG image of the body wall muscle shows the change from SB to DB structure of the sarcomeres. PSHG analysis: c) Post-surgical pixel-resolution mapping of myosin *θ_SHG_* at the muscle (color bar in degrees); d) *θ_SHG_* (mean±1 standard deviation) for the muscles in the region surrounding the cut and in the control region(white squares). The control region was selected in the adjacent muscular cell far away from the axotomy. Unpaired two tailed *t*- test (n = 200 pixels for both sets) yields p<0.001 (****) meaning that *θ_SHG_* mean is significantly different between the two regions. Arrows point to the place of the laser axotomy. Scale bar 10 µm.

**Figure 8:**
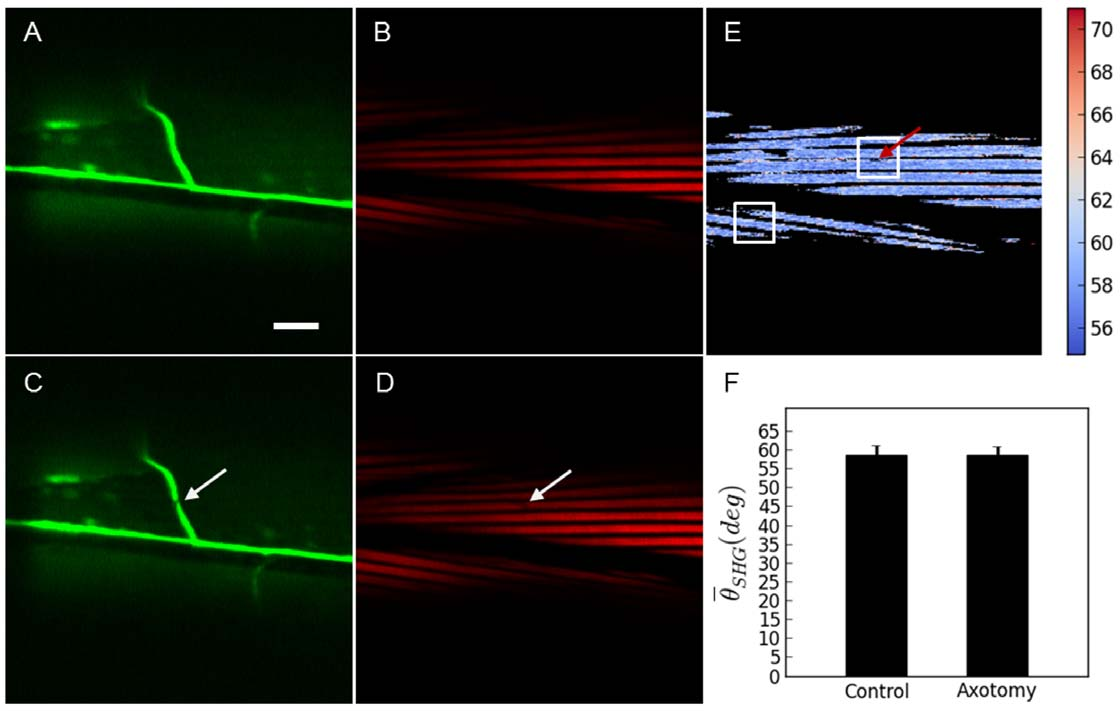
Comparison between PSHG and fluorescence for the muscle damage assessment of after the axotomy. Minimum collateral damage. Before the axotomy: a) TPEF image of YFP marked muscles and axons; b) SHG image of the muscles in the same region. After the axotomy: c) TPEF reveals a successfully cut axon, in the shape of a gap that interrupts the continuity of the axon, but no other damage is apparent; d) SHG image of the body wall muscle shows a small signal decrease at the targeted point on the axon. No other change or structural transformation is evident. PSHG analysis: c) Post-surgical pixel-resolution mapping of myosin *θ_SHG_* at the muscle (color bar in degrees); d) *θ_SHG_* (mean±1 standard deviation) for the muscles in the region surrounding the cut and in the control region(white squares). The control region was selected in the adjacent muscular cell far away from the axotomy. Unpaired two tailed *t*- test (n = 200 pixels for both sets) yields p>0.05 meaning that *θ_SHG_* mean is not significantly different between the two regions. Arrows point to the place of the laser axotomy. Scale bar 10 µm.

The characterization of the damage that can be reported with the SHG technique was compared with a set of experiments using worms expressing YFP in the body wall muscles (other strains with different muscle labeling strategies could have also been used for this same purpose [11]). To be able to properly target the neuron axon with the laser, the worms were also expressing CFP in the D-Type motoneuron axons. In all these cases fluorescence, SHG and PSHG were addressed in the same axotomized ROI. Firstly, a large collateral damage, evidenced by a big fracture of a thick filament fiber, was produced (see Figure 6). Here, in both, the fluorescence and SHG images, a black region, is observed. This can be interpreted as the collateral damage. Then, a reduced collateral damage in which no evident muscle fiber is broken was selected. In this case, TPEF from the axons (CFP labeled) reveals a successfully cut showing a clear gap that interrupts the continuity of the axon. However, fluorescence from the muscle (YFP labeled) structure does not reveal any collateral damage. In contrast, SHG imaging shows (Figure 3) the appearance of Double-Band structure in each of the thick filaments around the axotomized region (see Figure 7). Upon analysis with the PSHG technique (unpaired two tailed *t*- test (n = 200 pixels for both sets)) we found that the region around the axotomy revealed significant differences (p<0.001 (****)) when compared to an intact sarcomere region. Finally, we analyzed a case in which TPEF from the axons (GFP labeled) reveals a successfully cut axon (shown as a gap that interrupts the continuity of the axon) but no other damage was apparent. Here a highly confined (with a few pixels) decrease of SHG signal near the axotomized neuron was observed (see Figure 8). In this case, there was no Double-Band structure being produced and our PSHG analysis (unpaired two tailed *t*- test (n = 200 pixels for both sets)) did not find any significant difference (p>0.05) with intact tissue. This suggests that no damage has been produced or that this has been propagated in a negligible way after the axotomy procedure, as it is the case of Figure 5.

Table 1 summarizes the total number of successful axotomy experiments and the techniques used for the damage assessment. The potential structural damage on the muscle cells in the “NO apparent damage” set of results could only be done with PSHG imaging. This means that the 9 experiments that show disruption of the SHG signal, and potential damage in the muscular nano-organization, would have been overseen without the use of this technique.

**Table 1:** Successful axotomies and associated damage assessment techniques.

Successful axotomies (n = 56/61)	Method/Observation (n)
NO apparent damage (12)	PSHG: Revealed NO Myosin modification (3)
	PSHG: Revealed myosin modification (9)
Damaged (44)	Confocal, TPEF: damage is revealed through autofluorescence (33)
	Transmitted light: Observation of long lasting bubbles in the cuticle (4)
	SHG: Lack of the characteristic SHG signal in muscle due to fiber disruption (44)

This table summarizes the total number of successful axotomies (n = 56 out of 61) and the techniques used for the damage assessment.

## Discussion

In this study we employed a MHz femtosecond laser oscillator to precisely sever axons of *C. elegans* D-type motoneurons. The axotomies were recorded in real-time (*i.e*. during the axotomy process) using a set of linear and nonlinear imaging techniques implemented in a multimodal microscopy workstation. Pre- and post-surgical high resolution images were used to further assess the collateral damage caused to the surrounding tissues. TPEF and SHG provided the anatomical references to precisely locate the laser spot on the target axon. SHG and PSHG microscopy were used *a posteriori* to determine the collateral damage inflicted to the muscle whereas LT was used for the damage assessment of the cuticle.

Laser axotomy and multimodal imaging were performed on 61 different axons. Of these, 56 resulted in a successful cut, with or without some degree of collateral damage. As we set the power of the laser close to the threshold for axon cutting, smaller and more confined collateral damage was produced. The typical sizes of collateral damage ranged between 1 and 3 microns if fluorescence was used for the assessment (with some exceptions as the one in the Figure 6 that extended over 10 microns). SHG images reported damages from small punctures sizing less than a micron up to 10 microns when the sarcomere fibers were cut (see Figure 6). In both cases the lower limit of detection is given by the resolution limit of the employed methods (close to 0.5 microns in confocal, TPEF and SHG).

The variation in the induced damage is inherent to the fact that the technique is applied to a biological sample. Each worm used in the experiments, although carefully selected and synchronized, is different and can be positioned in different ways causing, therefore, different types of “errors” and different degrees of efficiency of the cut (and induced damage).

This is also why an efficient and versatile tool for collateral damage detection is needed. By the use of our multimodal imaging tool we were able to identify that in ∼20% of the cases (12 neurons) no collateral damage whatsoever could be detected. It is interesting to note that by using only increased autofluorescence (widely accepted metric) as an indicator of damage, this number would have been ∼54% (33 neurons). In such a case, this would not account for the minute damage produced along the axial extension of the focused beam and, therefore, its effects in the cuticle and/or the body wall muscles that do not show up as autofluorescence.

Previous collateral damage studies have been done based on: i) axonal regeneration speed and efficiency [5] [12], ii) distance between the sealed axon endings and monitoring of the muscle damage using a fluorescence reporter [11] and iii) the behavioral mechanism associated with a certain kind of neurons [20]. Methods that rely only on the generation or the suppression of the fluorescence do not discriminate between different tissues and can be affected by other fluorescent phenomena associated to the axotomy, such as axoplasm spilling and autofluorescence generated by stimulation of the cells [4]. The other methods, however, do not provide a direct visualization, do not detect minute collateral damage caused to the surrounding tissues nor are strong and robust indicators of the induced cut (such is the case of the distance between sealed axons as this varies with natural retraction of the axon tips during surgery).

Likewise, in regeneration studies, the effectiveness of functional reconnection of both axon endings could also be biased by having damage in the surroundings of the axons and possibly interfering with media guidance cues. On the other hand, the methodology here described results in a precise way for assessing this damage that is independent from any post-surgical factors. It includes a stringent quantitative criteria based on observation of the several indicative factors of collateral damage such as i) induced autofluorescence in the borders of the target area or in neighboring tissues, ii) cuticle damage, iii) muscle modification and iv) cavitation bubbles. This could be used in a wide range of biological applications in which *in vivo* damage assessment after laser nano-surgery is required, including neuronal regeneration studies.

Furthermore, in this work we have introduced, for the first time, the SHG imaging technique for determining collateral muscle damage after axon surgery. SHG/PSHG allows inferring the muscular structures at the molecular level (myosin) [21]–[22] and has, therefore, the potential to reveal minute collateral damages to the muscle. Although the relevance of these minute damages is not obvious for the axon regeneration process, this study presents a quantitative method that would allow a proper assessment of such influences.

In our experiment we have observed several different changes induced to the muscles: punctures in the muscle, reduction in SHG signal and change of the muscular fibre structure. Punctures are the result of direct collateral damage induced to the muscle mediated by the same physical mechanisms responsible of the axon dissection. In the case of a reduction of the SHG signal with no transition from SB to DB, our PSHG analysis revealed that there is no damage of the muscle at the myosin level (as this is the molecule generating the SHG signal). On other hand, when SB to DB conversion happens, this can be easily quantified with the PSHG technique. This suggests that there is an important impact on the myosin structure due to the axotomy.

Finally, structural changes such as a wave-like pattern appearance and SB to DB conversion have been seen to also occur together with a strong stretch/contraction of the muscle (see Media S2) [4]. Previous studies have related the change from SB to DB pattern to chemical degradation of *ex vivo* samples [23] and laser induced photo-damage [24]. In this work, we employed living *C. elegans*, indicating that this conversion is not related to fixation chemicals. Also, the laser powers employed for non-linear imaging were set to the level considered safe [25]. Therefore, both simultaneous SB to DB conversion and wave-like pattern formation can be seen as a side effect of the localized high-power laser irradiation employed to cut the axons.

Our results show that femtosecond pulses from our mode-locked laser oscillator can be effectively used to perform high precision neuron surgery *in vivo* with controlled collateral damage. The beam had an average power of 90 mW (an energy per pulse of 1.2 nJ) which are focused with an objective lens of 1.4 NA. This corresponds to a peak intensity of approximately 3×10^12^ W/cm^2^. Under these conditions, the ablation is mediated by dissociative effects of the generated low plasma density, in conjunction with photochemical effects into the irradiated volume. It was shown that in this regime, smooth nanometer-scale dissections can be obtained [1] [26]–[27]. Moreover, we can assume that the maximum damage is confined to the volume where the plasma is created. In our case, considering the theoretical estimations of the PSF of the focused beam with our experimental parameters we obtained a plasma distribution with dimensions 222 nm transversal and 613 nm axial. Therefore, even if the laser is perfectly focused at the axial center of the axon, damage in either cuticle or body wall muscles is expected, as can be seen in Figure 1. Furthermore, a small mistake on the axial positioning of the laser scalpel will be revealed as an increased damage whether in the muscle or in the cuticle. Fortunately, our method is capable to detect and account for any of these effects by using the proposed multimodal damage assessment technique.

## Conclusion

This study shows that femtosecond pulses from a mode-locked laser oscillator can be effectively used to perform high precision nano-surgery and provides an alternative approach for a practical and comprehensive damage assessment. This method has the potential to be used in most neuronal structures in *C. elegans* since there are many cases where different neuronal processes (not only axons but also dendrites) have neighboring muscles and cuticle. Most head neurons in *C. elegans* such as the sensory neurons, for instance, that make up a high percentage of the neurons in this model organism that are thoroughly used for behavior studies, are highly attractive candidates due to their strategic position around the pharynx, a big muscular structure that can be easily visualized using SHG microscopy. The application of the above mentioned techniques can be extended to other biosamples containing different endogenous sources of SHG (myosin, collagen, microtubules, etc.). The use of combined imaging techniques in one imaging set-up is an unconventional approach to *in vivo* studies of different structures and tissues that can prove to be an extremely valuable tool to the examination of induced damage by laser surgery tools.

## Supporting Information

Media S1 **Linear techniques for damage assessment.** Axotomy process in *C. elegans.* LT shows damage to the cuticle, while confocal imaging shows the induced autofluorescence and the retraction of the cut ends of the axon.
(AVI)

Media S2 **Axotomy with no apparent damage.** The axotomy is performed and the only evident effect is the interruption of the continuity of the axon. Nevertheless, other alterations, such as contraction and relaxation of the muscle cell can be observed.
(AVI)
